# Anesthetic management of Platypnea–orthodeoxia syndrome in a young patient with residual atrial septal defect following congenital heart surgery

**DOI:** 10.1186/s40981-025-00844-2

**Published:** 2025-12-24

**Authors:** Yoshifumi Naito, Misao Yoshikawa, Michiyo Yamano, Takeshi Nakamura, Fumimasa Amaya

**Affiliations:** 1 Department of Anesthesiology, Kyoto Prefectural University of Medicine, 465 Kajii-Cho, Kamigyo-Ku, Kyoto, 602-8566 Japan; 2 Department of Cardiovascular Medicine, Kyoto Prefectural University of Medicine, 465 Kajii-Cho, Kamigyo-Ku, Kyoto, 602-8566 Japan

**Keywords:** Platypnea-orthodeoxia syndrome, Atrial septal defect, Right-to-left shunt, Agitated saline contrast echocardiography, Paradoxical embolism

## Abstract

**Background:**

Platypnea–orthodeoxia syndrome (POS) is a rare condition causing dyspnea and hypoxemia that worsen in the upright position and improve when supine. While often reported in elderly patients, POS in young adults, particularly due to residual atrial septal defects (ASD) after congenital heart surgery, is uncommon.

**Case presentation:**

We present a 24-year-old woman with POS caused by a residual ASD following congenital heart surgery. Agitated saline contrast transthoracic echocardiography confirmed marked right-to-left shunting exacerbated by positional change and Valsalva maneuver.

Anesthetic management focused on minimizing shunt flow by maintaining systemic vascular resistance, avoiding hypoxia, hypercarbia, and acidosis, and carefully adjusting ventilation parameters. Transesophageal echocardiography was utilized for shunt evaluation and device placement. Additionally, continuous cerebral oximetry was monitored for possible ischemic changes associated with paradoxical embolisms. The patient underwent successful percutaneous ASD closure without complications.

**Conclusion:**

This rare case of young-onset POS highlights the importance of understanding the dynamic shunt physiology and vigilant intraoperative monitoring to ensure patient safety.

**Supplementary Information:**

The online version contains supplementary material available at 10.1186/s40981-025-00844-2.

## Background

Platypnea–orthodeoxia syndrome (POS) is a rare condition characterized by dyspnea and worsening hypoxemia in the upright or seated position, which improves in the supine position. Its etiology is typically due to a right-to-left shunt or ventilation-perfusion mismatch [1]. POS is typically described in elderly individuals with aortic dilatation or spinal deformity. In contrast, young-onset cases after congenital heart surgery are extremely uncommon. We report the anesthetic management of a young woman with POS caused by a residual atrial septal defect (ASD) after arterial switch operation, highlighting perioperative strategies to prevent right-to-left shunt exacerbation and ensure patient safety.

## Case presentation

**Fig. 1:**
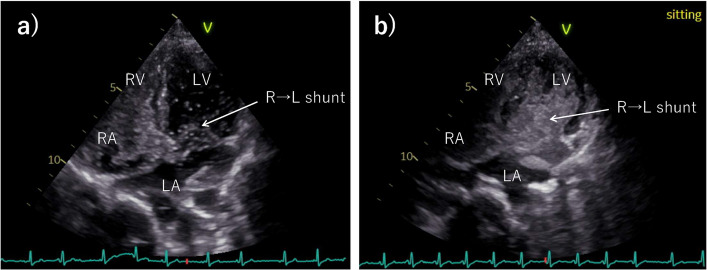
Agitated saline contrast (ASC) transthoracic echocardiographic findings in the supine and sitting positions. In the supine position, the ASC study revealed a small right-to-left shunt, whereas in the sitting position, the shunt was markedly increased. RV, right ventricle; RA, right atrium; LV, left ventricle; LA, left atrium; R → L, right to left

A 24-year-old woman (height, 162 cm; weight 43 kg) underwent an arterial switch operation for false Taussig–Bing anomaly during the neonatal period. Since adolescence, she had experienced recurrent fatigue and presyncope, which were often triggered by standing or prolonged activity. During her clinical training as a nurse, she developed fatigue and desaturation to the 80% range. Multiple emergency evaluations showed normal oxygenation in the supine position, delaying diagnosis. Outpatient evaluation revealed SpO₂ decreasing from 97% supine to 92% upright. Transthoracic echocardiography (TTE) demonstrated accelerated right ventricular outflow tract (RVOT) flow (peak velocity > 1.8 m/s) and an ASD with right-to-left shunting accentuated in the sitting position on agitated saline contrast (ASC) imaging (Fig. 1). Based on these findings, POS due to a residual postoperative ASD was suspected, and the patient was admitted for further evaluation and management.

On admission, the patient’s SpO₂ reproducibly decreased by more than 5% from the supine to the sitting position. Laboratory tests revealed polycythemia (hemoglobin, 14.9 g/dL) and a mildly elevated brain natriuretic peptide level (25.3 pg/mL), while liver (AST, 23 U/L; ALT, 21 U/L) and renal function (creatinine, 0.66 mg/dL; eGFR, 90.73 mL/min/1.73 m^2^) were within normal limits. Electrocardiography demonstrated sinus rhythm with right axis deviation. Chest radiography showed a cardiothoracic ratio of 48% without cardiomegaly or pulmonary congestion.

**Fig. 2:**
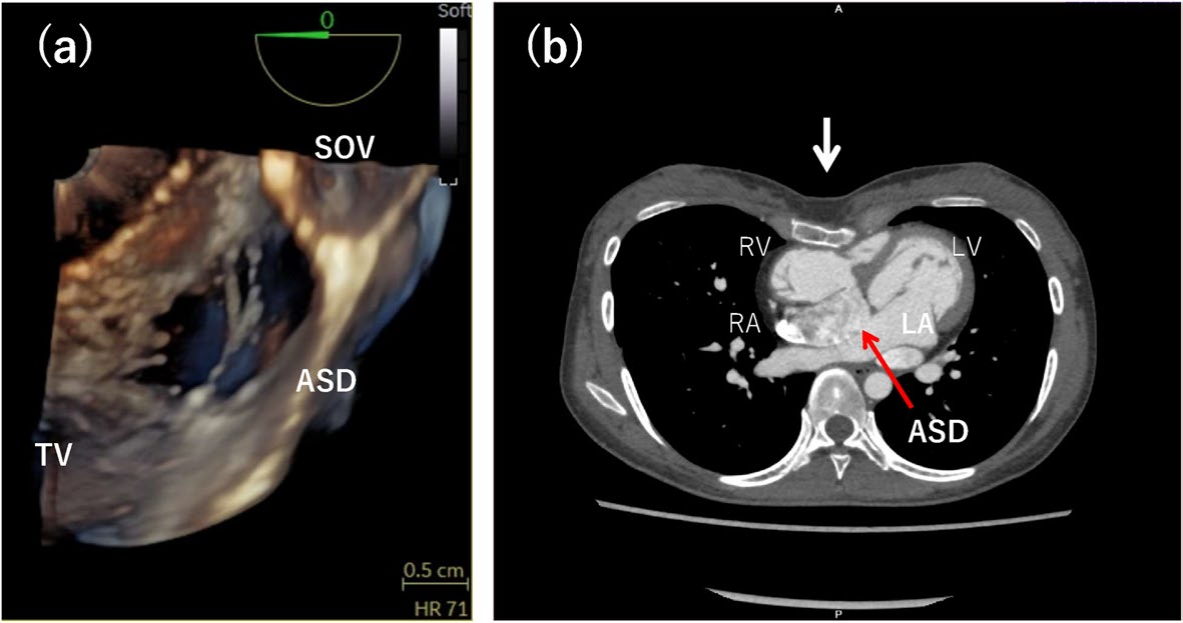
Imaging findings. **a** Three-dimensional transesophageal echocardiography (TEE) from the right atrial side showing an atrial septal defect (ASD) divided into three orifices by two septal structures. **b** Chest computed tomography showing an ASD (red arrow) and right ventricular compression due to sternal depression (white arrow). ASD, atrial septal defect; TV, tricuspid valve; SOV, sinus of Valsalva; RV, right ventricle; RA, right atrium; LV, left ventricle; LA, left atrium

Transesophageal echocardiography (TEE) demonstrated a predominantly left-to-right shunt at rest. However, ASC testing confirmed a small right-to-left shunt at rest, which increased in the sitting position. Three-dimensional TEE revealed a multi-orifice ASD (16.8 × 11.1 mm) with an almost absent aortic rim (1.5 mm) and a protruding sinus of Valsalva toward the interatrial septum (Fig. 2a). Cardiac CT demonstrated a 16-mm ASD and compression of the right ventricle due to sternal depression (Fig. 2b). Cardiac catheterization revealed a pulmonary-to-systemic blood flow ratio of 1.13 and a right-to-left shunt of 0.22 L/min (6.3%). Pulmonary vascular resistance was 1.15 Wood units. Right ventricular pressure measured 45/8 mmHg, pulmonary artery pressure was 28/8 mmHg, and there was an 18 mmHg gradient across the RVOT. Right atrial pressure was relatively elevated (right atrium: 12/2 mmHg; left atrium: 12/0 mmHg). Based on these findings, POS secondary to a residual postoperative ASD was diagnosed, and transcatheter ASD closure was scheduled.

**Fig. 3:**
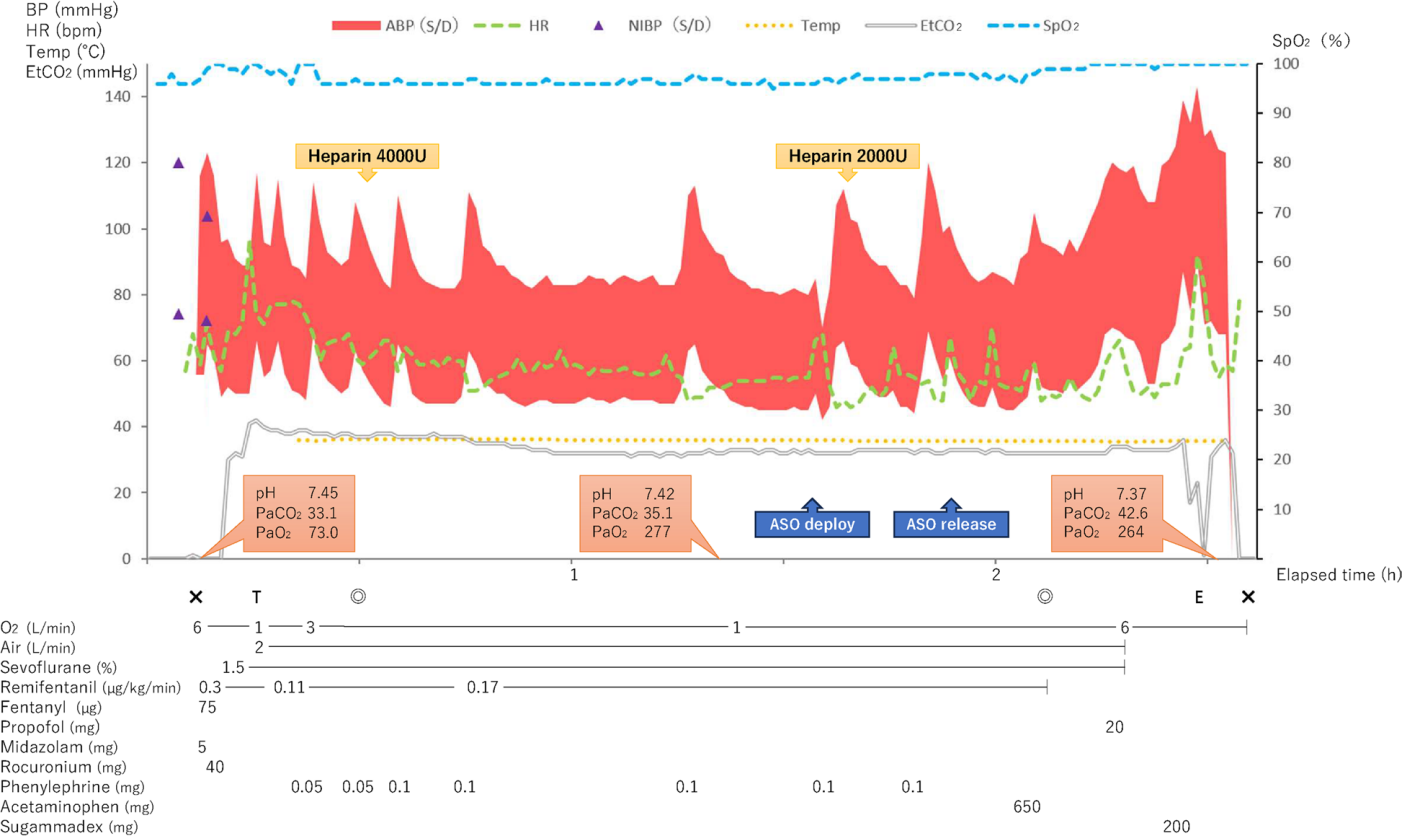
Intraoperative anesthetic record. Systolic/diastolic arterial blood pressure (ABP S/D), heart rate (HR), non-invasive blood pressure (NIBP S/D), body temperature (Temp), end-tidal CO₂ (EtCO₂), peripheral oxygen saturation (SpO₂) were continuously or intermittently recorded throughout the procedure. Arterial blood gas measurements are shown as pH, PaCO₂ (mmHg), and PaO₂ (mmHg). Time on the horizontal axis is shown in hours. Regional cerebral oxygen saturation (rSO₂) was continuously monitored during the procedure; however, it was not recorded in the electronic chart due to lack of integration. No decreases in oxygenation were observed. Events: X, start and end of anesthesia; T, tracheal intubation; E, tracheal extubation; ◎, start and end of surgery; ASO: Amplatzer™ Septal Occluder

**Fig. 4:**
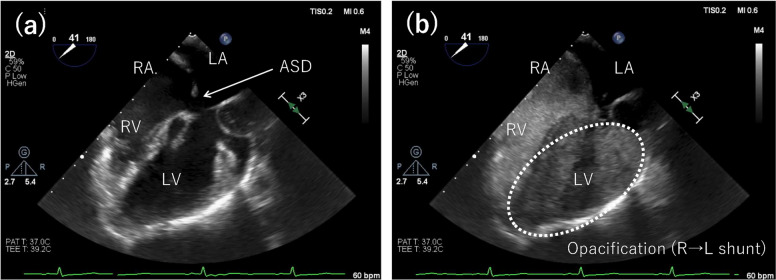
Agitated saline contrast (ASC) transesophageal echocardiography in the mid esophageal four chamber view during the modified Valsalva maneuver under general anesthesia. **a** Before ASC injection. **b** During the ASC study under general anesthesia with a modified Valsalva maneuver, the entire left ventricle was filled with contrast. RV, right ventricle; RA, right atrium; LV, left ventricle; LA, left atrium; R → L, right to left; ASD, atrial septal defect

**Fig. 5:**
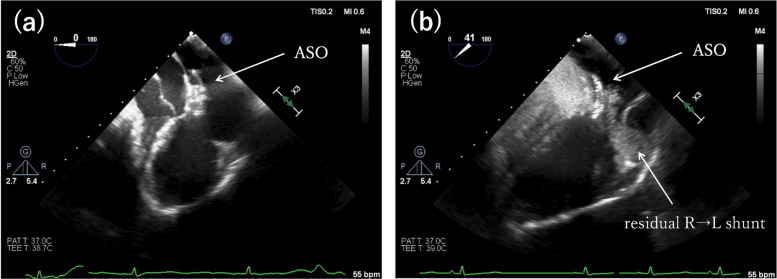
Agitated saline contrast (ASC) transesophageal echocardiography in the mid esophageal four chamber view during the modified Valsalva maneuver after transcatheter atrial septal defect (ASD) closure. To confirm a residual right-to-left shunt after transcatheter ASD closure, an ASC study was performed under a modified Valsalva maneuver. **a** Before ASC injection. **b** A small leak was observed; however, there was a marked reduction in the number of bubbles. ASO, Amplatzer™ Septal Occluder; R → L, right to left

General anesthesia was induced with midazolam, remifentanil, fentanyl, and rocuronium, and maintained with sevoflurane (Fig. 3). Invasive arterial pressure, TEE, and cerebral oximetry were continuously monitored. After sheath insertion via the right femoral vein, a total of 6000 U of intravenous heparin was administered to maintain activated clotting time > 250 s. The anesthetic goals were to minimize shunt flow and reduce the risk of paradoxical embolism. Phenylephrine was administered intermittently to maintain systemic blood pressure, and mechanical ventilation was set with a tidal volume of approximately 8 mL/kg and a PEEP of 4 cmH₂O to achieve normocapnia. Intraoperative TEE confirmed the morphology of the defects consistent with the preoperative findings. The ASC study demonstrated a right-to-left shunt, which increased further during a modified Valsalva maneuver, consisting of held expiration and release under general anesthesia (Fig. 4, see supplementary Video 1) [2]. The primary defect was closed using a 15-mm Amplatzer™ Septal Occluder (Abbott Medical, Plymouth, MN, USA). Post-deployment TEE confirmed device stability, minimal residual shunting, and no interference with the mitral valve (Fig. 5, see supplementary Video 2). No intraoperative hypoxemia or decrease in regional cerebral oxygen saturation was observed. Total intraoperative fluid administration was 900 mL, and the anesthetic duration was 2 h and 30 min. The patient was extubated in the operating room after emergence from anesthesia. Transient premature atrial contractions occurred post-procedure but resolved by the following day. Postoperatively, SpO₂ was maintained at 97–98%. The patient’s recovery was uneventful, and the patient was discharged the day after surgery. At the six-month postoperative follow-up, the patient’s SpO₂ was 100% in the sitting position, with complete resolution of the patient’s work-related symptoms.

## Discussion

POS is defined as a postural decrease in PaO₂ of more than 4 mmHg or a decrease in SpO₂ exceeding 5% [1]. Although POS is reported more frequently in elderly patients, its onset in young individuals, as in the present case, is rare. The primary pathophysiology involves hypoxemia due to increased right-to-left shunting, which is exacerbated by postural changes through the interplay of anatomical and physiological abnormalities [3]. POS can be classified into intracardiac and extracardiac types. The intracardiac POS, the right-to-left shunt across an interatrial communication is most commonly due to a patent foramen ovale, but also reported with an ASD or atrial septal aneurysm [4]. Two mechanisms have been proposed for shunt exacerbation. The first is anatomically redirection of right atrial blood flow toward the left atrium without elevated right atrial pressure. This mechanism has been associated with aortic root dilatation, a prominent Eustachian valve, and thoracic or spinal deformities that predispose to flow redirection toward the atrial septum during postural changes [5, 6]. The second mechanism involves increased right atrial pressure, resulting in an augmented interatrial pressure gradient, as seen in right ventricular myocardial infarction, pulmonary embolism, pulmonary hypertension, constrictive pericarditis, or pericardial effusion [6, 7].

In extracardiac POS, conditions such as pulmonary arteriovenous malformations, hepatopulmonary syndrome, or ventilation–perfusion mismatch in the lower lung fields due to pulmonary fibrosis or chronic obstructive pulmonary disease may exacerbate shunting when upright because of increased basal pulmonary perfusion [1, 8].

In this case, the patient had a residual ASD following surgery for congenital heart disease, without evidence of pulmonary pathology, suggesting an intracardiac POS. Chest CT revealed a sternal depression compressing the right ventricle, leading to elevated right heart pressures and accelerated RVOT flow. A modified Valsalva maneuver during TEE provoked a marked right-to-left shunt, implying that transient increases in right atrial pressure during postural changes enhanced the shunt. Furthermore, deviation of the atrial septum by a protruding sinus of Valsalva and absence of the aortic rim likely redirected blood flow into the left atrium. These findings indicate that combined anatomical and hemodynamic mechanisms contributed to POS development.

For the diagnosis of POS, comparing SpO₂ in supine and upright positions is essential. If oxygenation is assessed only in the supine position, postural hypoxemia may be overlooked even in young individuals. In intracardiac POS, both TTE and TEE are highly valuable [9]. A right-to-left shunt is diagnosed if ASC appears in the left atrium within three cardiac cycles after injection [10]. TEE is particularly useful for evaluating shunt direction and volume with postural change. Although positional assessment under general anesthesia can be technically challenging, a modified Valsalva maneuver, as in this case, can reproduce comparable hemodynamic effects.

Anesthetic management of patients with intracardiac POS should focus on preventing exacerbation of right-to-left shunting. Severe hypoxemia can result from decreased systemic vascular resistance and hypovolemia, both of which may lower left ventricular end-diastolic pressure and left atrial filling, thereby increasing right-to-left shunt flow even without postural change [11]. The marked increase in shunt flow during the Valsalva maneuver in this case also suggested that POS, unlike typical ASD, is vulnerable to hemodynamic changes, which may trigger right-to-left shunting even in the supine position. In addition, factors that elevate pulmonary vascular resistance—such as hypoxia, hypercapnia, and acidosis—should also be avoided as they can raise right heart pressures and further worsen the condition. Maintenance of appropriate anesthetic depth, judicious vasopressor use to prevent excessive reductions in systemic vascular resistance, and careful fluid management to preserve preload are recommended [12]. In this patient, systemic vascular resistance was maintained with intermittent vasopressor administration, and ventilator settings were adjusted to prevent hypercapnia or increased airway pressure.

The risk of paradoxical embolism must also be carefully addressed in patients with right-to-left shunting. Coronary air embolism during ASD closure has been reported [13], highlighting the importance of meticulous air elimination from all intravenous lines and sheaths. Positive-pressure ventilation may increase right heart pressures, further elevating the risk of air embolism; thus ventilator settings, such as positive end-expiratory pressure, should be carefully optimized [14].

TEE is invaluable for confirming device position, residual shunting, and detecting paradoxical embolism in real time. In this case, significant right-to-left shunt exacerbation was confirmed with a modified Valsalva maneuver during TEE, guiding anesthetic management to minimize shunt flow. Continuous cerebral oximetry provides an additional non-invasive tool for the early assessment of changes in cerebral oxygenation associated with embolic events. Although it cannot directly detect microemboli, it enables timely identification of cerebral hypoperfusion or ischemia. Previous studies have highlighted that ischemic changes due to emboli may not be detected if they occur outside the monitored region, which represents a limitation of rSO₂ [15]. Therefore, complementary monitoring with transcranial Doppler can enhance the identification of embolic events and improve overall safety.

Although no complications occurred in this patient, successful anesthetic management of POS during transcatheter closure requires understanding of the hypoxemia mechanism, dynamic assessment of shunt flow during postural changes or the Valsalva maneuver, careful perioperative management to avoid shunt exacerbation, and meticulous monitoring for the prevention and early detection of paradoxical embolism. Given the limited literature on anesthetic care in POS, this case provides valuable insights that may inform future perioperative care in similar scenarios.

## Supplementary Information

Supplementary Material 1. Agitated saline contrast transesophageal echocardiography (ASC-TEE) performed before Amplatzer^TM^ Septal Occluder (ASO) placement, under a modified Valsalva maneuver.

Supplementary Material 2. Agitated saline contrast transesophageal echocardiography (ASC-TEE) performed after Amplatzer^TM^ Septal Occluder (ASO) placement, under a modified Valsalva maneuver.

## Data Availability

The supplementary videos supporting this case report are available in the online version of this article. Other datasets are not publicly available due to patient privacy.

## Abbreviations

POS: Platypnea–orthodeoxia syndrome
ASD: Atrial septal defect
SpO₂: Percutaneous oxygen saturation
TTE: Transthoracic echocardiography
RVOT: Right ventricular outflow tract
ASC: Agitated saline contrast
AST: Aspartate aminotransferase
ALT: Alanine aminotransferase
eGFR: Estimated glomerular filtration rate
TEE: Transesophageal echocardiography
CT: Computed tomography
PaO_2_: Arterial oxygen partial pressure
